# Experiences and the psychosocial situation of parental caregivers of children with spinal muscular atrophy against the background of new treatment options: a qualitative interview study

**DOI:** 10.1186/s40359-024-02070-4

**Published:** 2024-10-17

**Authors:** Maja Brandt, Joenna Driemeyer, Jessika Johannsen, Jonas Denecke, Laura Inhestern, Corinna Bergelt

**Affiliations:** 1 Department of Medical Psychology, the University Medical Center Hamburg-Eppendorf, Martinistraße 52, Hamburg, 202446 Germany; 2 Department of Pediatrics, the University Medical Center Hamburg-Eppendorf, Martinistraße 52, Hamburg, 202446 Germany; 3 Department of Medical Psychology, University Medicine Greifswald, Walther- Rathenau- Straße 48, Greifswald, 17475 Germany

**Keywords:** Spinal muscular atrophy, Psychosocial, Caregiver burden, Supportive care needs, Qualitative, Spinraza®, Zolgensma®

## Abstract

**Background:**

Spinal muscular atrophy is a rare neurodegenerative disorder in children which leads untreated to muscle wasting, respiratory impairments, and a shortened life expectancy. Parents as primary caregivers are often physically and psychologically burdened. In recent years, new and promising treatment options have been approved, but it remains unclear if they have an impact on the psychosocial situation of affected families.

**Objectives:**

The aim of this study was to explore the views and experiences of parents as informal caregivers of children with SMA in the course of the disease against the background of new treatment options (Spinraza® or Zolgensma®).

**Methods:**

We conducted qualitative interviews with 27 parents of children with SMA treated with Spinraza® and Zolgensma® from April to September 2020. The analysis was done using thematic analysis and reported according to the COREQ criteria.

**Results:**

The data analysis resulted in three main themes: a) caregiver burden and negative consequences for families, b) resources and protective aspects, c) psychosocial care needs. The results are discussed against the background of new treatment options and previous models of supportive care needs. Parental caregivers of affected children face multiple burdens in different stages of the child’s disease progression.

**Conclusion:**

Although new treatment options for SMA showed observable effects for most parents, the main caregiver burden and reported symptoms were attributable to the overburdening care tasks. To unburden families, more screening for unmet needs, family-centered help services, professional caregivers, childcare, and sufficient financial support are needed.

**Supplementary Information:**

The online version contains supplementary material available at 10.1186/s40359-024-02070-4.

## Background

### Spinal muscular atrophy (SMA)

Spinal muscular atrophy (SMA) is a rare neurodegenerative condition that usually begins in infancy, resulting in muscle wasting and weakness, and depending on its severity, shortens life expectancy and impairs the motor abilities as well as the ability to breathe [1–3]. It is caused by a homozygous disruption of the surviving motor neuron 1 (SMN-1) gene by deletion, conversion, or mutation [1]. Until recently SMA was one of the most common genetic diseases causing infant mortality with an incidence of approximately one in 10 000 live births and a carrier frequency of one in 50 [1, 2].

In the natural course of the disease, SMA is divided into clinical subtypes related to the highest acquired motor function and the age at symptom onset. The severity of the disease is divided into clinical subtypes that correlate with the age of onset and the number of remaining copies of the surviving motor neuron 2 (SMN-2) gene:

SMA type 1 (accounts for about 50–60% of all SMA cases) is the most severe and occurs in children under six months of age. Children affected by this type are unable to sit unassisted and have an expected survival time of no more than two years without treatment or respiratory support [1, 2, 4, 5].

Type 2 SMA (30%) occurs in children between 7 and 18 months of age. Untreated, affected children usually learn to sit but not to stand or walk, have respiratory support needs, and often die in their adolescent years [1].

Type 3 (10%) occurs after 18 months of age and shows a wide range in symptom heterogeneity. It is characterised by the ability to walk (with support) and normal life expectancy, but patients show progressive orthopaedic impairments, e.g., like joint contractures and scoliosis [1, 2, 4, 6].

To diagnose SMA, patients who show typical clinical symptoms must be tested for the homozygous deletion or mutation of the SMN-1 gene and SMN-2 copy number [1]. Due to the rarity of SMA, initial symptoms are often misjudged as developmental delays by parents and paediatricians, leading to diagnostic delays of up to several years in the past [7]. Therefore, to detect and treat children in a pre-symptomatic stage, SMA has been included in new-born screening (NBS) on a pilot basis since 2018 and integrated into regular testing in some European countries, as well as in the USA, Canada, and Australia since 2020 [8, 9]. In Germany all new-borns are tested for SMA since July 2021 [9].

### Changes in treatment options for SMA

Until 2017, standard care for SMA mainly included palliative care, (non-)invasive ventilation and gastrostomy for severely affected patients, and scoliosis surgery and orthotic care for all types of SMA [2, 5]. Within the past years, three new disease-modifying medical treatment options (nusinersen (Spinraza®), authorised in 2017, onasemnogene abeparvovec-xioi (Zolgensma®), authorised in 2020 and risdiplam (Evrysdi®), authorised in 2021 for use in the European Union) have been successfully approved for clinical use [10, 11].

Although existing treatment options can reduce the mortality and severity of disease progression in symptomatic patients, curing SMA is still not possible and the long-term efficacy remains unclear [4, 10]. A recent review of 12 months follow-up of one of the three new treatment options or a combination of different options showed improvements in motor skills for type 1 patients and stabilisation in deterioration in type 2–4 for the treatment with Spinraza®, Zolgensma® or a combination of both. However, according to the authors, there is a lack of long-term clinical data beyond pivotal studies published by independent clinicians, and the heterogeneity of studies hinder their comparability, leaving open questions about long-term disease stability and persistence of gained capabilities [12]. Another recent review of the efficacy of Evrysdi® showed improved motor function in patients with SMA types 1, 2 and 3 for up to 2 years. Although Evrysdi® provides a useful treatment option across a broad range of ages and subtypes of SMA, according to the authors further data are required to better determine its place in the management of SMA [13].

The treatment of pre-symptomatic children showed even more promising results: children with type 1 SMA treated with Zolgensma® pre-symptomatically showed rapid age-appropriate achievement of motor milestones and improvements in motor function, indicating the benefit of early treatment [14].

However, treatment effects and clinical approval of treatment options depend on the age and clinical characteristics (e.g., number of copies of the SMN-2 gene) of patients and, due to the high cost of treatment, on the coverage of the respective country, which makes access to treatment and treatment decisions complex for affected families [15].

### The role of parents as informal caregivers in children with SMA

The literature on the impact of SMA on parents as informal caregivers implicates multiple sources of burden, as well as negative psychological, social, and economic effects for affected families: Existing studies on quality of life, economic and social burden in affected families show that most of the parents of children with SMA report a high financial burden, a high amount of caregiver hours per day (≥ 10 h), reduced working hours for paid work and an impaired quality of life [16–19].

In a recent systematic review of quantitative and qualitative studies of the psychosocial and caregiver burden of children and adolescents with SMA [20], conducted by our research group, most of the 27 studies reported reduced levels of quality of life and moderate to high levels of caregiver burden and distress, as well as physical and mental health symptoms in caregivers. This was especially true for families whose children had more severe subtypes of SMA (type 1–2). Further, findings indicate several unmet family needs regarding information, care coordination, treatment decisions, financial support, and adequate supportive care services.

However, only two of the 27 included studies in the review were conducted with samples, in which children received one of the new treatment options for SMA [20]. A more recent German study examining the psychosocial burden of parents 12 months after the positive NBS result for SMA showed a high burden of disease in parents, regardless of whether they had already pre-symptomatically opted to treat their child with Spinraza® or Zolgensma®, with even higher rates of “parental worries” in the treatment group [21]. This might suggest that new treatment options or the introduction of NBS do not necessarily unburden affected families, but the parents' view of their psychosocial life situation since the introduction of new treatment options is still understudied. This leaves open the question of how parents of children with SMA who receive one of the new treatment options experience their care situation, what their main stressors and resources are, and what psychosocial support needs arise against the background of the new treatment options.

## Methods

### Objectives of this study

The aim of this study was to explore the views and experiences of parents as informal caregivers of children with SMA in the course of the disease against the background of new treatment options (Spinraza® or Zolgensma®). We examined aspects of psychosocial and caregiver burden of parents, protective factors, and resources as well as psychosocial supportive care needs of families.

### Participants and recruitment

Participants were selected with purposive sampling [22, 23] which selects participants “with particular characteristics who will better be able to assist with the relevant research” and who has “knowledge and experience (…) and the ability to communicate experiences and opinions in an articulate, expressive, and reflective manner” [23].

To be included, participants had to be a parent or legal guardian of a child or children affected by SMA and to have enough language skills to understand and answer the researchers’ questions. Further, we included parents whose children were under the age of 18 years, had a confirmed diagnosis of SMA type 1–3, and who currently received Spinraza® or Zolgensma® treatment in the Department of Neuropaediatrics in the University Medical Center Hamburg-Eppendorf (Germany). Patients did not necessarily have to be diagnosed at the University Medical Center and could be treated by other healthcare professionals (HCPs) before or in addition to the administration of medication. For those patients who had been diagnosed in the Department of Neuropaediatrics, standard of care included 2–3 appointments per 90 min to discuss the diagnosis with the parents.

Parents with insufficient language skills or who were too burdened to participate in our study (self-assessment) and parents not providing informed consent were excluded. Further, we excluded parents whose children were already deceased. We interviewed all eligible parents, who gave their informed consent, between April and September 2020 during the stay of their child or children in the hospital for one of the treatments (Spinraza® or Zolgensma®) face to face or, if parents wished so, within the following 14 days after the treatment via telephone. We stopped recruiting after no more new topics emerged within the interviews.

The first author (MB), who does not belong to the staff of the Department of Neuropaediatrics, was contacted by the treating physician when families came to the hospital for the planned treatment with Spinraza® or Zolgensma®. Families were then contacted during their stay in the hospital or via telephone to be educated about the study content and objectives and to invite one parent (father or mother), who accompanied the child for the treatment, for the interview. We contacted 34 parents, of which six were excluded because of insufficient language skills and five parents declined to participate because of too much psychological burden. In total, 23 mothers and four fathers participated. Table 1 shows the characteristics of participants.

**Table 1:** Characteristics of participants

Variable	n	%
Parents/ Family characteristics	27	
Female	23	85
Male	4	15
Age (M, SD)	36.8 (5.2)	
School education/ Family status		
≤ 10 years	13	48
11–13 years	14	52
Living in a partnership	23	85
Lone	4	15
Number of children living in the family (M, SD)	1.9 (0.9)	
Employment status		
Full-time homemaker	8	30
Paid parental leave	6	22
Full-time worker	3	11
Part-time worker	10	37
Patient characteristics	28	
Female	19	68
Male	9	32
Age (M, SD)	6.7 (4.6)	
SMA type		
Type 1/intermediate type 1–2	9	32
Type 2/intermediate type 2–3	16	57
Type 3	3	11
Diagnosis via new-born screening (NBS)	1	4
Time since start of symptoms until diagnosis in months (M, range)	8.25 (0.5–42.0)	
Children born and diagnosed before clinical approval of Spinraza® (July 2017)	17	61
Treatment option		
Spinraza® (nusinersen)	25	89
Zolgensma® (onasemnogene abeparvovec)	1	4
Both	2	7
Respiratory support		
Non-invasive	12	43
Invasive	1	4
Tube feeding		
Percutaneous endoscopic gastrostomy (PEG) tube	7	25
Nasogastric tube	1	4
Number of aids and appliances (M, range)	6.3 (0–15)	

The mean age of children was 6.6 years (range: four months to 14 years), 19 of 28 children were girls, most children had SMA type 2 or intermediate type 2–3. Seventeen children (61%) had been born and diagnosed before the approval of Spinraza® for clinical use in Germany (July 2017). Further, only one child (4%) was detected and treated pre-symptomatically via NBS. Most children received Spinraza® (89%), only few received Zolgensma® treatment or had received both treatments. Twelve children (43%) received non-invasive respiratory support; one child received invasive ventilation. Eight children (29%) needed tube feeding. Further, children needed on average 6.3 assistive devices and appliances like wheelchairs or other medical or therapeutic equipment on a daily basis.

### Data collection

A semi structured interview guide was developed by the researchers after reviewing the existing literature and was adapted in discussion with the currently treating physicians. For interview topics and questions, see Table 2. 22 interviews were conducted face-to-face and five via telephone. The mean length of interviews was 32 min (range: 13–48 min). All interviews but two were conducted by the first author (MB, female), the rest was conducted by another member of the research team (DZ, male). Both interviewers (MB, DZ) are psychologists in an advanced stage of therapy training and had experience in conducting qualitative interviews for research. All interviews were conducted in German, the audio was recorded after consent of the interviewees and later transcribed verbatim. Key quotations from the parents were later translated into English by the first author (MB). We reported our methods and findings according to the Consolidated criteria for reporting qualitative research (COREQ; for details, see Additional file 1 in the supplemental material) [24].

**Table 2:** Interview topics and questions

Introductory questions: sociodemographic and medical characteristics	
• Age	• Family situation/marital status
• Country of birth	• Number and age of children
• School education	• SMA type and progression of child
• Employment status	• Childcare of affected child
Interview topic guide	
1. Diagnosis	• Process of diagnosis
	• Communication of diagnosis by physician
	• Experience of receiving diagnosis/ period of time
2. Child’s treatment	• Experience of medical treatment and perception of treatment effects with new treatment option
	• Experience of aids and appliances and of daily care
3. Impact of SMA on the parent/ family	• Impact of disease on different life aspects (work, finances, family members, relationships, social life, life goals)
	• Effects on caregiver’s physical and mental health
4. Perceived support and supportive care needs	• Resources and perception of support
	• Use of support services
	• Supportive care needs (after receiving the diagnosis, in the course of treatment)

### Coding and analyses

**Fig. 1:**
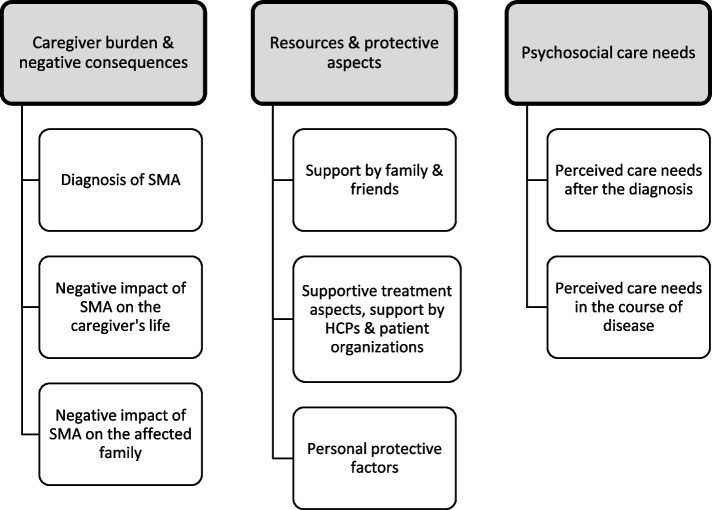
Thematic map of main and subthemes emerging from the interviews with parents as informal caregivers of a child affected by SMA

The thematic analysis (TA) approach [25] was used to identify key concepts within the participants’ perspectives. The coding and analysis process conducted using a deductive-inductive coding using the six phases of reflexive TA suggested by Braun and Clarke [25, 26] as guideline for the coding process: Firstly, two members of the research team (MB, SH) read the material and made notes about their opinion on relevant issues presented in the data. Secondly, the first author (MB) developed initial codes and searched for themes (step 1–3), which were discussed with the second person familiar with the data (SH). Thirdly, the first author reviewed the initial themes, created a thematic map (Fig. 1) and defined themes (step 4–5), which were discussed in the research group. Finally, all authors checked and compared the research question with the coded material and contributed to the production of the report (step 6). We used MAXQDA version 2020 to manage the data. For the detailed code system, see Additional file 2 in the supplemental material.

### Ethical considerations

The study was approved by the local psychosocial ethics committee (LPEK-0174). All participating parents gave their written informed consent.

## Results

The data analysis resulted in the identification of three main themes: a) perceived caregiver burden and negative consequences for families, b) resources and protective aspects, c) perceived psychosocial care needs (see Fig. 1).

### Perceived caregiver burden and negative consequences for families

#### Diagnosis of SMA

Most participants described receiving the SMA diagnosis as a shock and traumatic event, accompanied by symptoms like feelings of numbness, narrowing of consciousness, limited attention, and disorientation, causing high emotional burden, which sometimes lasted for several months, sometimes even years to process.

*“It was a kind of trauma, (…) I can't really describe it, as if I hadn't experienced it myself and as if it wasn't real. It took quite a while.” (I23, mother of a child, SMA type 1)*

The feeling of shock and trauma was even worse if the communication of the diagnosis or education about the disease was perceived as too direct, not compassionate enough by the person who delivered the diagnosis, or if too little time was taken for the conversation. In addition, the diagnosis was experienced very negatively and increased the parents' fears if the prognosis given focused too much on the shortened life expectancy and too little on opportunities and treatment options.

*“It was bad. They [HCPs] just slapped it on you like that between the door and the corner: ‘Your child might die in a month, if you're lucky in three years’, something like that. (…) Then you said goodbye inside, you weren't told what the possibilities were, what chances the children had, nothing, you only had the internet.” (I3, mother of a child, SMA type 2)*

Besides the upcoming fears of the future and uncertainty about how long their affected child will live, some parents further described they had no time to process the diagnosis and felt left alone with the sudden need to organize the next steps of care for their child.

*“There is no one to do it for you or to tell you that you must go here or there. You have to somehow do everything yourself. That was difficult in the beginning.” (I16, mother of a child, SMA type 2)*

#### Negative impact of SMA on the caregiver’s life

Most participants described that their lives changed drastically after their child's diagnosis: Parents caring for a child with SMA, especially those affected by the more severe forms of SMA 1–2, more often described being highly burdened, for example, spending many hours on caregiving tasks and care coordination day and night.

*„For me, the most stressful thing is the care, that you somehow manage everything during the day. We make a timetable so that we can tick off everything and then not go crazy ourselves (…)”. (I12, mother of a child, SMA type 1)*

Especially care coordination, including applications for assistive devices and appliances, as well as financial claims, organizing treatment appointments, and finding suitable ambulatory therapists, as well as options for suitable childcare and schooling, was perceived as exhaustive and time consuming. Further, care pathways were often described as opaque and bureaucratic and some parents had to fight with health insurances refusing to meet the costs for i.e., assistive devices.

*“(…) you really must fight for a lot of things, make phone calls, work it out, somehow it is not made clear to you what you are entitled to. (…) it has been a miserably long way.” (I17, mother of child, SMA type 1–2)*

In addition, the regular hospital visits for the administration of Spinraza® were predominantly experienced as time-consuming and emotionally burdening. In particular, the high treatment frequency (usually 4 administrations in the first 2 months, thereafter every 4 months), the length of hospital stays (2 nights), the child’s fears and pain before or during the procedure (lumbar puncture (LP)) were described as hard to bear.

“*I find it stressful that we must be here for three days, that we have to plan it, also with our infant [sibling] at home, that one of us [parents] has to leave our job, even though I have employers who totally support me. But still, sometimes I find all this organizing very annoying.” (I8, mother of child, SMA type 2)*

For some parents, the hospital visits were additionally stressful if they perceived a lack of continuity in staff, a lack of time or a lack of focus on their child's needs and feelings.

*“I come from a nursing background myself and sometimes I think ‘Guys, these are little children, they are not machines, of course they express their fears differently than we do!’. So sometimes I've clashed with some people [HCPs], (…) because at some point I have to defend him [child].” (I14, mother of a child, SMA type 2)*

As a result of high caregiver burden, participants described overburdening and lack of free time, often leading to emotional strain and in a deterioration of mental and physical health.

*“So, the stress level has increased. I had to deal with panic attacks because the stress level was too high, so my general practitioner said, keep the stress level up for four more weeks and you'll be crawling on all fours, you can't do that anymore.” (I17, mother of a child, SMA type 1–2)*

Further, parents who were the main caregiver of the affected child (mostly mothers), described drastic changes in their working life due to the caregiving situation: most participants gave up their work, reduced their working hours, have been terminated due to work absence for the child’s care or lack of professional caregivers. This was especially problematic for single parents.

*“In some cases, we had no nursing service at all for half a year, which means I can't go to work. But the employer won't go along with that either. So now I'm completely at home and must see if I can find a job again at some point.” (I3, mother of a child, SMA type 2)*

Most work adjustments due to the caregiving situation resulted in loss of income, causing financial strain.

*“(…) there [at work] is no more understanding [for the child’s illness] and that is why my husband’s contract was not extended because of poor performance, (…) which means that now my husband is unemployed, and I am on parental leave (…). That is an extreme financial crash compared to our situation three years ago.” (I23, mother of a child, SMA type 1).*

#### Negative impact of SMA on the affected family

Participants reported that SMA not only affected their life as primary caregiver, but also had a substantial negative impact on the life as a family. For instance, the child’s symptoms, the care tasks, and care coordination (e.g., treatment appointments) dominated the organization of the whole family. If the affected child was unable to walk or sit, parents had to find barrier-free leisure time activities, holiday destinations and housing options, which were mostly reported to be complicated to organize.

*“You are still limited everywhere. (…) it already starts in the planning, what is suitable for the disabled child? (…) do we take the wheelchair? That restricts everything.” (I3, mother of a child, type 2)*

Further, participants reported that previous life plans had to be abandoned due to the uncertainty of disease progression, and that they only plan in the short-term or not dare to make plans at all.

*“You plan differently, you plan more cautiously and not for many years (…). Now we know, okay, she will stay with us longer, it makes sense [to plan].” (I17, mother of a child, type 1–2)*

Some parents described changes in the family’s social life like social withdrawal. The reasons were mostly lack of time due to care tasks, avoiding the risk of lung infections of the affected child, or differences in lifestyle compared to families with healthy children.

*“When you have a disabled child, you live a completely different lifestyle than neighbours who have children of the same age, they have a different daily routine, we had to structure it differently.” (I19, mother of a child, type 2).*

Also, the relationships within the family changed in the course of the disease: Most parents reported having little or no time anymore to spend with their partner or spouse, because the intensive care tasks and economic situation of the family forced them to divide their roles as the main caregiver (mostly the mother), and the main breadwinner (mostly the father). Even if parents could spend time together as a couple, they reported being too exhausted or that at least one parent had always to stay with the affected child.

*“My husband always works from morning to night and then I'm basically responsible for everything, for everyday life, and you're just exhausted after the day.” (I4, mother of a child, type 1–2)*

As a result, some parents who were the main caregiver reported that they felt not enough supported by their partner, had more conflicts with their partner or had the feeling of drifting apart from each other.

*“You really have to be careful that you don't start living side by side, we already have the problem that everyone goes his own way, but togetherness falls apart.” (I17, mother of a child, SMA type 1–2)*

Besides changes in the partnership, parents often reported disadvantages for healthy siblings: Firstly, after taking care of the affected child, parents did not have much time or energy left to take care of the needs of their other children. Secondly, older siblings were often involved in supporting their parents with care tasks or babysitting or had to be considerate of the disability of their affected sibling. Thirdly, siblings were emotionally burdened too if the disease of the affected child progressed.

*“The big one takes care of her like a second mum, she's totally into it, but she's also sad, her grades at school have dropped.” (I22, mother of a child, SMA type 2)*

Further, some parents report a lack of support from their families of origin or conflicts with the child’s grandparents, aunts, or uncles about his or her condition.

*“On my husband's side, unfortunately, the family dealt with it [SMA-diagnosis] very badly. (…) my mother-in-law, with whom we basically lived under one roof, said from the beginning: ‘I can't take her, I can't help you’, so my mother had to come from far away when something happened.” (I4, mother of a child, type 1–2).*

Besides the loss of income due to work adjustments for care tasks, parental caregivers reported further financial burdens for families due to required assistive devices and appliances, which were not covered by their health insurance, costs for barrier-free house alterations or the purchase of cars suitable for disabled people.

*“(…) a vibration plate, which is super important for therapy, it is not covered by the health insurance. It costs seven thousand euros, that's not just paid easily.” (I12, mother of a child, SMA type 1).*

About half of the parents who had contact with patient organisations or self-help groups reported that the contact had been more stressful for them than helpful. The main reason was that families perceived the contact with other affected families, who were not doing well or whose children were even more severely affected, as an additional burden. Others did not find the services suitable for their situation or had reservations about opening up to strangers.

*“(…) I always thought to myself, do I really want to burden myself with this, do I really want to see that children are worse off than my child with the same illness, I always said, no, I want to stay with myself somewhere.” (I9, mother of a child, SMA type 2)*

### Resources and protective aspects

Although all parents reported burden or negative impact of SMA on their life, most of them also reported supportive aspects, which helped them to cope with their child’s diagnosis and the consequences of it.

#### Support by family and friends

Most parents reported to receive their main support by their families of origin. The caregivers’ parents, especially their mothers or mothers-in-law, but also other relatives like fathers, siblings, or older children were helping either with support in everyday life (e.g., babysitting, household assistance) or with emotional or financial support.

*“My mother-in-law can't lift her from the wheelchair into bed or anything like that, but she can babysit her, play a game with her, (…) or simply having a sympathetic ear, if I have any worries or concerns (…).” (I17, mother of a child, SMA type 1–2)*

Further, the parent who was the main caregiver, often named her or his partner or spouse as biggest supporter with care tasks or emotional problems. Some parents reported that their partnership quality even improved as they experienced coping with their child's disease as bonding.

*“Our marriage has become rather better, fortunately, because my husband is very, very, very involved (…). He was very involved from the beginning; we are a good team for our daughter.” (I23, mother of a child, type 1)*

For some parents, friends were also important sources of support in addition to relatives and partners. Like relatives of the caregivers, friends were mainly mentioned as support in everyday life or as emotional support.

*“Our friends were really great! When we bought the house, they helped us with the renovation and the move. They were unbelievably normal with my daughter (…). They really helped us a lot.” (I4, mother of a child, SMA type 1–2)*

#### Supportive treatment aspects, support by health care professionals and patient organisations

Most parents stated that they had observed an improvement in symptoms (especially motor skills) due to the medication (usually Spinraza®), which some of them found emotionally relieving. In addition, the development of several new treatment options within the last few years gives some parents reason to hope that their child's development will be better than predicted or that the progression of SMA can be stopped one day.

*“The fact that there's a drug and we're also seeing progress means that we're very well provided for. Of course, we are looking forward to seeing what else there might be in the future. The other two drugs [for SMA] don't promise a cure (…), but maybe in ten years.” (I15, mother of a child, SMA type 2).*

Parents felt well supported by their child's health care team if their contact persons remained the same over the course of treatment, if physicians responded promptly to parental concerns, were empathetic in their contact with the family, and provided competent and sufficient advice to the family on questions and treatment decisions or organized further treatment if needed.

*“I feel that these two doctors are somehow part of our lives. I think they are almost like family members to us. (…) they have become two very important people in our path. I somehow totally trust them.” (I9, mother of a child, SMA type 2).*

Furthermore, about half of the participants stated that they felt relieved and supported by professional carers (e.g., nursing services, hospice services, respite care), therapists (e.g., physiotherapists, speech therapists), specialised childcare (e.g., school assistance) or domestic aids.

*“We have an outpatient hospice service that supports us in word and deed, that also helps us to look after the children, to get one's breath back, so that we can go for a walk or have some time and rest.” (I17, mother of a child, SMA type 1–2)*

Approximately half of the parents who had contact with patient organisations or self-help groups experienced the contact as helpful and perceived them as a source of information or for sharing experiences with other affected families.

*“You get information from families who have been doing this for much longer than we have and that is quite good. And let's not fool ourselves, the parents know better than any nurse or doctor.” (I10, mother of a child, SMA type 1)*

#### Personal protective factors

Some aspects that were protective for parents resulted from their personal situation and/or personality factors. For example, some parents did not have any financial problems despite loss of income or high additional costs due to SMA due to inheritance or good earnings of the working parent.

*“I probably won't be able to do my job anymore. In our case we don't have financial problems, because fortunately we are well supported by my family (…).” (I18, mother of a child, SMA type 2–3)*

Some main caregivers, mostly mothers, reported that their job is an important balance to the situation at home.

*“I totally love my job and I think it's a total shame that I can only work twelve hours because of my family situation, because (…) I just totally love my job.” (I9, mother of a child, SMA type 2)*

As important as a job to balance out the care work, for some parents it was essential to have enough time for themselves or to be able to pursue hobbies.

*“(…) I've already had the point where I really said, ‘I can't carry on much longer, I have to get out now!’. Then I decided for myself, I need a little something for myself. I've been making music for over thirty years, so I need that too.” (I25, mother of a child, SMA type 2–3)*

Some parents reported feeling less burdened if they had a certain positive attitude towards their life situation. This included, among other things, a certain mental stability, the ability to cope well with the child's illness, feeling positive about caring for the child, and feeling self-effective, for example, after the diagnosis.

*“I have to say that we have learned to deal with it [SMA] and to appreciate life. Because you can see what you can somehow get out of it. And because she [daughter] is in such a good mood and has so much fun, she also makes it quite easy for us.” (I15, mother of a child, SMA type 2)*

### Perceived psychosocial care needs

Along with the negative impact of SMA on the lives of affected families and their resources and protective factors to deal with these effects, parents also reported unmet psychosocial care needs in the course of the disease.

#### Perceived care needs after the diagnosis

Most parents expressed the need for more counselling and information after being informed of their child’s diagnosis, especially if they had been too shocked to recall information provided after hearing the diagnosis for the first time. This included information on entitlement to maintenance and how to apply for it, information on patient organisations and self-help groups, as well as information on how to live with SMA, what assistive devices or appliances are needed or what parents need to do in emergencies (i.e., respiratory arrest).

*“What am I entitled to? What can I apply for? To what extent can I apply for something that is also good for him? These are things that I always wonder about that I don't really know, and it's difficult to get information, especially from the health insurance companies (…)” (I14, mother of a child, SMA type 2)*

Beyond that, some parents wished for psychological support, especially after the diagnosis, but also on a long-term basis.

*“I think the first time here in the hospital was the worst, because we didn't get any psychological support. We asked several times, but no one came, and they said, there is no such thing as a crisis intervention service.” (I12, mother of a child, SMA type 1)*

One father expressed the wish to shorten the time to diagnosis for families to be able to start treatment earlier and thus improve the prognosis of affected children.

*“In the end, this would have allowed us to start treatment almost a year and a half earlier, perhaps, if it had been an attentive paediatrician, and that would of course have had a great effect on the course of the disease (…).” (I8, father of a child, SMA type 3).*

#### Perceived care needs in the course of disease

Regarding long term support needs in the course of the disease, most parents stated that they would like to get more support with and information about care and care-coordination, as this was the main part of their daily burden. This included, above all, the wish for more capacities of care and nursing services, specialised childcare provisions and mobile therapists who also came to the families' homes.

*“There should be some kind of possibility to take care of our daughter, I want to go to work, I don't want to live on social welfare, that's another hard step financially, but leaving the financial aspects aside, I have my job, I like to work in my job.” (I22, mother of a child, SMA type 2)*

Regarding care coordination, parents mainly wanted to get support in bureaucratic matters, such as applying for healthcare services, as well as in case and care management.

*“There would be a need for someone to coordinate everything [treatment] (…), it's so chaotic, the right hand doesn’t know what the left hand's doing and at the end of the day it's the child who suffers” (I12, mother of a child, SMA type 1)*

Moreover, some parents wished for more integrative care places (e.g., in centres for chronically ill children) and more schools adapted to the special needs of their children (i.e., barrier-free access, school assistance, integration of therapy services at school).

*“Grammar schools should please make sure that they are also disabled-accessible. (…) She's always been special anyway and now she's getting another spot because she can't go to the school where she'd like to, with her friends, just because she has a wheelchair (…).” (I25, mother of a child, SMA type 2–3)*

Regarding their child’s medical treatment, some parents further wished for closer medical supervision, permanent contact persons with a personal contact to the family, and to be closely involved in the treatment planning.

*“Before this nusinersen administration, there was always a quarterly outpatient doctor's appointment, which no longer exists. With the medication, I have a gap in my knowledge, which should be clarified again in a doctor's consultation. (…)” (I9, mother of a child, SMA type 2)*

## Discussion

### Caregiver burden and negative consequences

Our study aimed to investigate the psychosocial situation and experiences of parents caring for a child affected by SMA after the introduction of new disease-modifying treatment options (Spinraza®, Zolgensma®). The results showed multiple sources of burden and negative consequences if a child is affected by SMA, not only for parental caregivers but also for the family as a whole. Although our study was conducted after the introduction of new therapies, many children had been diagnosed before and already were symptomatic at the time of treatment. In our sample, only one child was detected via NBS, as screening had not yet been regularly implemented in Germany at the time of the interviews. Further, the majority (61%) of our sample was born and diagnosed before Spinraza® was approved in 2017, so these children often could not start treatment until later than clinically recommended. Both aspects may have a strong impact on treatment efficacy and prognosis.

Families of children affected by SMA types 1–2 with more severe symptoms and disease progression were of particular burden. Besides the shock of receiving a life-threatening diagnosis for their child, many parents described long-term physical and mental overburdening due to care tasks and care coordination. Due to lack of professional care services and flexible work possibilities, the main caregivers, mostly mothers, were forced to reduce working hours or quit their employment, which often had a negative impact for the mother’s and family’s financial situation and future. Further, parents had to split up in the roles of main caregiver and main breadwinner, leading to less family time, more conflicts, and disadvantages for healthy siblings. Even though some of them voluntarily reduced working hours or quit their employment to spend more time with their children at home, others described feeling forced to do so due to lack of professional care services. One way or the other, the main caregiving work and caregiver burden was mostly worn by mothers, reinforcing already existing gender imbalances regarding unpaid care work [27–29].

These results are consistent with other studies on the burden of caregivers and the psychosocial situation of affected families, which were carried out before new treatment options became available [20]. However, another recent study also suggests that the burden of parents whose children were diagnosed by NBS is high and does not differ between parents who opted for or against pre-symptomatic treatment of their child [21]. Therefore, it might also be possible that the burden of the diagnosis of SMA (e.g., the shock and uncertainty about the course of the disease and treatment) has not (yet) changed much for parents at an individual level, despite improvements in treatment options. This might suggest that the availability of new treatment options for SMA does not per se contribute to an improvement in the overall psychosocial situation of the affected families until more longitudinal studies have shown whether the new treatment options are able to improve the course of the disease as positively as hoped for a wide range of SMA types. Nevertheless, it is likely that we were not yet able to sufficiently reflect possible changes in the burden on families due to the timing of our survey shortly after the introduction of the new treatment options and the high rate of symptomatic patients with experience of diagnosis before the era of new treatment options. Therefore, more studies are needed with samples diagnosed via NBS and treated early to identify a possible change in psychosocial burden due to the introduction of the new treatment options.

Additionally, navigating the health care system is significantly more difficult for patients with rare diseases due to the rarity of their condition, making aspects such as health insurance coverage, childcare, and treatment decisions even more difficult than for non-rare diseases [30, 31]. Further, specifically in the case of SMA, with the introduction of new treatment options parents face more uncertainty regarding treatment decisions and their impact on disease progression [32].

### Resources and protective aspects

As main resource of support, most parental caregivers named their partners and other family members (like grandparents, siblings, aunts, and uncles of the affected child), less named friends of the family as support. For parents, the most helpful support was help with daily and care tasks, babysitting, emotional or financial support. Further, a stable financial situation of the family and enough free time to pursue hobbies were reported to be beneficial. Especially parental caregivers without a partner or family support nearby reported to be more burdened and restricted in their employment situation and therefore also financially. This implies that families without a sufficient social support system and especially single parents are disadvantaged, reinforced by the lack of outpatient care services. This is in line with previous studies, which found that caregivers with less perceived social support had higher caregiver burden, higher stress and were at higher risk of developing depression [33, 34].

Regarding the medical treatment of the affected child, parents reported to feel well supported, if the treatment team of the child remained constant, showed empathy and interest for the child and family and were available, when the family needed something from them. The new treatment options, most notably Spinraza®, gave parents hope for the future course of disease of their child, especially when they saw improvement in their child’s motor abilities. This is in line with the findings of our review, which found hope to be a central theme in several qualitative studies where children were treated with Spinraza® [20]. A more recent Canadian qualitative study on the experiences of informal caregivers with Zolgensma® treatment also found hope to be a central theme [35]. However, at the same time, parents also reported fear and uncertainty as central, which was attributed to the novelty and uncertainty of the long-term effects of Zolgensma® at the time of the survey. This could also be an indication of why parents who had decided in favour of or against Zolgensma® treatment for their child showed a similarly high level of burden [21]. Therefore, factors such as time of diagnosis (before/after symptom onset) and available treatment options may influence the feeling of hope and further studies are needed to investigate the aspect of hope in relation to caregiver burden and treatment decision-making.

Even though the medical treatment with Spinraza® was seen as positive by most parents due to observable treatment effects, it was not as prominent in the interviews and did not seem to drastically change the whole family’s situation. This might suggest that as long as medical treatment options for SMA are not (yet) able to stop the disease progression or cure the disease, the overall impact on the family’s life remains rather low. However, as mentioned above, this might also be attributable to the characteristics of our sample (diagnosis and treatment with mostly Spinraza® only after symptom onset or medication approval) and calls for further research. Indicating that, as recommended [15], treatment should start as early as possible, in a best way before the onset of symptoms.

### Perceived care needs after the diagnosis and in the course of disease

After receiving the diagnosis, many parents reported that they were not able to receive further information about the next steps due to the shock, leaving many questions about further treatment and care steps unanswered. In addition, some parents expressed the desire for psychological support after receiving the diagnosis in hospital and/or in the long term. Studies with informal caregivers in other chronic diseases (e.g. cancer) have shown that psychological interventions, especially psychological counselling, can contribute to a reduced burden of care [36]. To minimise the barriers to accessing psychosocial support, it is recommended that psychologists and social workers be included as part of a multiprofessional treatment team.

In the course of the disease, most parental caregivers in our sample expressed the wish for more support with the child’s care and care coordination, ranging from receiving more information (like addresses) to getting practical help by HCPs. This included help with daily care tasks by professional care services as well as help with bureaucratic matters for coverage of treatment expenses. This is similar with findings of a review of supportive care needs of parental caregivers caring for a child with a rare disease by Pelentsov and colleagues from 2015 [37] and the recent review by our research group specifically addressing SMA [20]. Both reviews included studies conducted before the clinical approval of any new treatment options and showed that most parents wished for either more information about the disease and helpful addresses for support or wished for direct support by HCPs with care and care coordination. However, even with the new treatment options, patients still require long-term medical and multidisciplinary care and care coordination, which is why support for caregivers in these aspects is still recommended [38]. This underlines the importance of a more psychosocial and family-centred treatment approach.

**Fig. 2:**
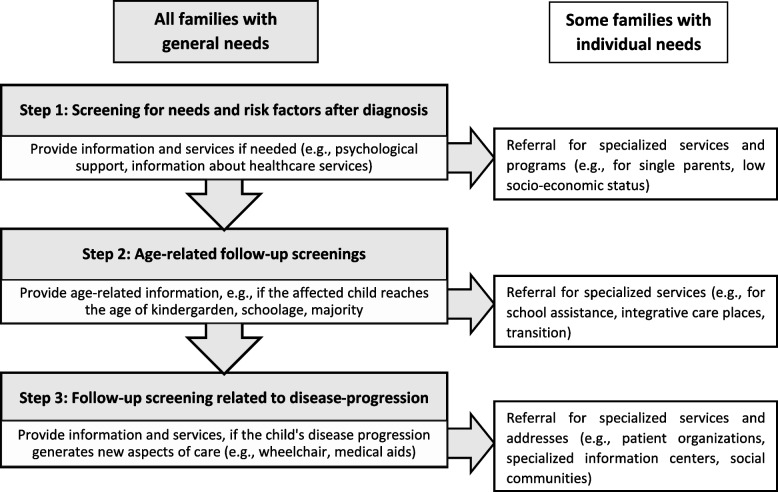
The Psychosocial Care Needs Model for SMA. A model of stepwise screening for supportive care needs for families affected by SMA at different points in child development or disease progression

Since supportive care needs might differ individually and not solely depend on symptoms and treatment received, HCPs should regularly assess psychosocial and supportive care needs of families proactively and enable them to find the appropriate support offers if necessary. To identify families in need for further support, it might be useful for HCPs to adapt the ideas of stepwise screening for supportive care needs in cancer patients, e.g., like included in the Tiered Model of Supportive Care by the Supportive Cancer Care Victoria or The Supportive Care Pathway [39]. Both models might be a useful approach for families affected by SMA to create a systematic and stepwise screening process in different stages of the child’s development and disease progression (e.g., after diagnosis, before school enrolment etc.) against the background of the family’s social situation (e.g., single parents, healthy siblings etc.). We have adapted the idea of stepwise screening for families affected by SMA, included SMA-specific time points and aspects based on the expressed needs by parental caregivers in our sample and summarized it in a model, the Psychosocial Care Needs Model for SMA (Fig. 2). This model can be used by HCPs to decide when a screening of psychosocial care needs of families might be useful and which support is needed. For details, see Fig. 2.

However, this screening process and approach can only succeed, if HCPs (e.g., paediatricians, social workers) have enough resources and if appropriate family-oriented services for different needs are available. Further, the expressed lack of professional care services by parents is a well-known healthcare problem within Germany and the EU [40, 41] and must be addressed on a more general level in health policies.

### Limitations

Our study has some limitations we need to address: Firstly, most children received Spinraza®, only few children received (also) Zolgensma® and none of them Evrysdi®, which was not yet approved for treatment in Germany during our data collection. Due to our study design, the novelty of the therapy options and the heterogeneity of age, onset, and severity of clinical symptoms of our sample of children, we cannot draw general conclusions if or how new therapy options changed the psychosocial situation of families, or the experiences of parental caregivers compared to earlier findings. In addition, all new medical options have the best treatment prognosis when the affected children are pre-symptomatic [15], which was only the case for one child in our study sample. This might have a big impact on the parents’ reflections on prior experiences especially on obtaining the diagnosis, when their child has been diagnosed before 2017. Therefore, more research with affected children born after the clinical approval and detected via NBS will be needed in the future.

Secondly, our sample was recruited from a single centre in northern Germany, which treats rare disease patients supra-regionally but is not representative of all parental caregivers. Further, our data collection was conducted during the Covid-19 pandemic. Even if the pandemic had no influence on the intervals and realisation of treatment appointments for the Spinraza® administration, it certainly changed the (perception of the) psychosocial burden, which some parents mentioned in the interviews (e.g., the impact of social isolation, fear of the impact of Covid on the course of the disease, etc.).

Thirdly, most interviewees were mothers. Although mothers might represent most parental caregivers, our findings might not be representative of the supportive care needs of fathers as parental caregivers.

Lastly, only one child in our study has been diagnosed with SMA via new-born screening (NBS), as it was not yet a standard in Germany at the time of our data collection [9]. As mentioned above, this might affect the experience of receiving the diagnosis of SMA for parents and should therefore be considered in future research.

## Conclusion

Parental caregivers of children affected by SMA are burdened in multiple ways and in different stages of the child’s disease progression. Although new treatment options for SMA showed observable effects for most parents and gave them hope for their child’s future, the main caregiver burden and psychological symptoms in our sample remained due to the overburdening daily care tasks, reinforced by the lack of outpatient care services, care coordination and already existing social disadvantages (female gender, single parenthood, low income). However, due to the timing of our data collection and the characteristics of our sample (mostly diagnosed before the availability of new treatment options and treatment after symptom onset), it is possible that changes in the overall burden have not been (fully) reflected in our study, which is why further studies are needed.

Since the overall burden and supportive care needs might differ individually and not solely depend on symptoms and treatment received, treatment centers should routinely integrate screening for supportive care needs and ensure addressing individual needs of both children and parental caregivers concurrent to the child’s medical treatment to support families affected by SMA. Additionally, to reduce families’ burden, health policies should enable more specialized family-centered help services, outpatient care services, childcare, and sufficient financial support as well as reduce administrative barriers. Further research is needed to inquire if and how the newly implemented new-born screening and the newly available pre-symptomatic treatment options have an impact on the psychosocial situation of families affected by SMA.

## Supplementary Information

Additional file 1. COREQ Checklist for reporting qualitative research.

Additional file 2. Code System with themes, sub-themes, and key quotations.

## Data Availability

The data supporting the results of this study are based on text excerpts translated into English by the authors. All excerpts relevant to the data analysis can be found within the manuscript or in the supplementary information material. Due to the sensitivity and identifying features contained in the original transcripts, these cannot be made publicly available. Nevertheless, it is possible to obtain anonymised excerpts of the data upon a reasonable request to the authors.

## Abbreviations

SMA: Spinal muscular atrophy
NBS: New-born screening
LP: Lumbar puncture
HCP: Healthcare professional
