# Supplementary material for: Experiences and the psychosocial situation of parental caregivers of children with spinal muscular atrophy against the background of new treatment options: a qualitative interview study
Source: BMC Psychol. 2024 Oct 17;12:566. doi: 10.1186/s40359-024-02070-4 (PMC11488358; doi:10.1186/s40359-024-02070-4)
Supplement: Supplementary file 2 — Additional file 2. Code System with themes, sub-themes, and key quotations. [file 40359_2024_2070_MOESM2_ESM.pdf]

Additional file 2 – Code System with themes, sub-themes and key quotations

Themes	Quotes[a]
**1. Perceived caregiver burden and negative consequences for families**	
1.1. Diagnosis of SMA	
Diagnosis as a shock or traumatic event	• *"It was a kind of trauma, (...) I can't really describe it, as if I hadn't experienced it myself and as if it wasn't real. It took quite a while." (I23, mother of a child, SMA type 1)*
Communication by HCPs worsens perception of diagnosis if performed too direct, too negatively focused (prognosis), not compassionate enough or if time of conversation was too short	• *"It was bad. They [HCPs] just slapped it on you like that between the door and the corner: 'Your child might die in a month, if you're lucky in three years', something like that. (…) Then you said goodbye inside, you weren't told what the possibilities were, what chances the children had, nothing, you only had the internet." (I3, mother of a child, SMA type 2)*
Fears and uncertainty about the future	• *"What really bothers me is that it's so uncertain what will happen now. Because really, from death to basically anything can happen and that makes it all so unpredictable. You don't really know what you can do." (I7, mother of a child, SMA type 1)*
No time to process the diagnosis and feeling left alone	• *"There is no one to do it for you or to tell you that you must go here or there. You have to somehow do everything yourself. That was difficult in the beginning." (I16, mother of a child, SMA type 2)*
1.2. Negative impact of SMA on the caregiver's life	
Drastic changes due to care and high caregiver burden	• *„For me, the most stressful thing is the care, that you somehow manage everything during the day. We make a timetable so that we can tick off everything and then not go crazy ourselves (…)". (I12, mother of a child, SMA type 1)*
Exhaustive and time-consuming care coordination	• *"It's quite time-consuming when you have a child with a disability like this, you have to constantly apply for medical aids, keep appointments, make phone calls to health insurance companies, make appointments with medical supply stores and doctors, there's no time to work on the side." (I17, mother of child, SMA type 1-2)*
Opaque and bureaucratic care pathways	• *"(...) you really must fight for a lot of things, make phone calls, work it out, somehow it is not made clear to you what you are entitled to. (...) it has been a miserably long way." (I17, mother of child, SMA type 1-2)*
Time-consuming and emotionally stressful hospital visits (for nusinersen administration)	• *"I find it stressful that we must be here for three days, that we have to plan it, also with our infant [sibling] at home, that one of us [parents] has to leave our job, even though I have employers who totally support me. But still, sometimes I find this organization very annoying." (I8, mother of child, SMA type 2)*
Overburdening, physical, and mental health problems	• *"So, the stress level has increased. I had to deal with panic attacks because the stress level was too high, so my general practitioner said, keep the stress level up for four more weeks and you'll be crawling on all fours, you can't do that anymore." (I17, mother of a child, SMA type 1-2)*

Drastic changes in working life (e.g., reduced hours, leaving jobs) and lack of professional carers	• *"In some cases, we had no nursing service at all for half a year, which means I can't go to work. But the employer won't go along with that either. So now, I'm completely at home and must see if I can find a job again at some point." (I3, mother of a child, SMA type 2)*
Loss of income and financial strain	• *"(…) there [at work] is no more understanding [for the child's illness] and that is why my husband's contract was not extended because of poor performance, (…) which means that now my husband is unemployed, and I am on parental leave (…). That is an extreme financial crash compared to our situation three years ago." (I23, mother of a child, SMA type 1)*
1.3. Negative impact of SMA on the affected family	
Organizing the family around the child's condition and care (e.g., holidays, activities, housing)	• *"You are still limited everywhere. (…) it already starts in the planning, what is suitable for the disabled child? (…) do we take the wheelchair? That restricts everything." (I3, mother of a girl, type 2).*
Rejection of earlier plans for life	• *"You plan differently, you plan more cautiously and not for many years (…). Now we know, okay, she will stay with us longer, it makes sense [to plan]." (I17, mother of a girl, type 1-2)*
Changes in the family's social life and social withdrawal	• *"When you have a disabled child, you live a completely different lifestyle than neighbours who have children of the same age, they have a different daily routine, we had to structure it differently." (I19, mother of a girl, type 2)*
Changes in partnership (e.g., few time, more conflicts)	• *"My husband always works from morning to night and then I'm basically responsible for everything, for everyday life, and you're just exhausted after the day." (I4, mother of a girl, type 1-2)*
Changes in family relationships (e.g., negative impact on healthy siblings)	• *"The big one [older sister] takes care of her like a second mum, she's totally into it, but she's also sad, her grades at school have dropped." (I22, mother of a girl, SMA type 2)*
Additional financial burdens due to care-related purchases (e.g., medical aids, cars)	• *"(…) a vibration plate, which is super important for therapy, it is not covered by the health insurance. It costs seven thousand euros, that's not just paid easily." (I12, mother of a girl, SMA type 1)*
Burdensome contact with patient organisations and self-help groups	• *"(…) I always thought to myself, do I really want to burden myself with this, do I really want to see that children are worse off than my child with the same illness, I always said, no, I want to stay with myself somewhere." (I9, mother of a boy, SMA type 2)*
***2.* Resources and protective aspects**	
2.1. Support by family and friends	
Caregiver's family as support in everyday life, as emotional or financial support	• *"My mother-in-law can't lift her from the wheelchair into bed or anything like that, but she can babysit her, play a game with her, (…) or simply having a sympathetic ear, if I have any worries or concerns (…)." (I17, mother of a girl, SMA type 1-2)*
Partner as supporter/ improvement of partnership quality	• *"Our marriage has become rather better, fortunately, because my husband is very, very, very involved (…). He was very involved from the beginning; we are a good team for our daughter." (I23, mother of a girl, type 1)*

Friends as support in everyday life and as emotional support	• *"Our friends were really great! When we bought the house, they helped us with the renovation and the move. They were unbelievably normal with my daughter (…). They really helped us a lot." (I4, mother of a child, SMA type 1-2)*
2.2. Supportive treatment aspects, support by health care professionals and patient organisations	
Emotional relief through treatment effects with new therapies	• *"We were quite happy with the treatment because she made progress relatively quickly, so we saw that it worked. Then we also felt a bit better." (I12, mother of a girl, SMA type 1)*
Hope for future drug developments	• *"The fact that there's a drug and we're also seeing progress means that we're very well provided for. Of course, we are looking forward to seeing what else there might be in the future. The other two drugs [for SMA] don't promise a cure (…), but maybe in ten years." (I15, mother of a child, SMA type 2)*
Consistent, empathetic, and reliable healthcare professionals	• *"I feel that these two doctors are somehow part of our lives. I think they are almost like family members to us. (…) they have become two very important people in our path. I somehow have total trust in them." (I9, mother of a child, SMA type 2)*
Support by professional carers and therapists (e.g., nursing and hospice services, physiotherapists, school assistance)	• *"We have an outpatient hospice service that supports us in word and deed, that also helps us to look after the children, to get one's breath back, so that we can go for a walk or have some time and rest." (I17, mother of a child, SMA type 1-2)*
Helpful contact with patient organisations and self-help groups	• *"You get information from families who have been doing this for much longer than we have and that is quite good. And let's not fool ourselves, the parents know better than any nurse or doctor." (I10, mother of a child, SMA type 1)*
2.3. Personal protective factors	
Wealth or stable financial situation (e.g., inheritance, good earnings)	• *"I probably won't be able to do my job anymore. In our case we don't have financial problems, because fortunately we are well supported by my family (…)." (I18, mother of a child, SMA type 2-3)*
Job as a balance to care tasks	• *"I totally love my job and I think it's a total shame that I can only work twelve hours because of my family situation, because (…) I just totally love my job." (I9, mother of a child, SMA type 2)*
Leisure time and pursuing hobbies	• *"(…) I've already had the point where I really said, 'I can't carry on much longer, I have to get out now!'. Then I decided for myself, I need a little something for myself. I've been making music for over thirty years, so I need that too." (I25, mother of a child, SMA type 2-3)*
Mental stability and/or positive attitude towards their life situation (e.g., acceptance, feeling positive towards care work)	• *"I have to say that we have learned to deal with it [SMA] and to appreciate life. Because you can see, what you can somehow get out of it. And because she [daughter] is in such a good mood and has so much fun, she also makes it quite easy for us." (I15, mother of a child, SMA type 2)*

***3.* Perceived psychosocial care needs**	
3.1. Perceived care needs after the diagnosis	
Need to receive more counselling and information (e.g., addresses)	• *"What am I entitled to? What can I apply for? To what extent can I apply for something that is also good for him? These are things that I always wonder about that I don't really know, and it's difficult to get information, especially from the health insurance companies (…)" (I14, mother of a child, SMA type 2)*
Psychological support (after the diagnosis and long-term)	• *"I think the first time here in the hospital was the worst, because we didn't get any psychological support. We asked several times, but no one came, and they said, there is no such thing as a crisis intervention service." (I12, mother of a child, SMA type 1)*
Shortening the diagnostic pathway (before the implementation of NBS)	• *"In the end, this would have allowed us to start treatment almost a year and a half earlier, perhaps, if it had been an attentive paediatrician, and that would of course have had a great effect on the course of the disease (…)." (I8, father of a child, SMA type 3)*
3.2. Perceived care needs in the course of disease	
Wish for more support with and information about care and care-coordination (e.g., more capacities of care and nursing services)	• *"There should be some kind of possibility to take care of our daughter, I want to go to work, I don't want to live on social welfare, that's another hard step financially, but leaving the financial aspects aside, I have my job, I like to work in my job." (I22, mother of a child, SMA type 2)*
Support for bureaucratic matters, case and care management	• *"There would be a need for someone to coordinate everything [treatment] (…), it's so chaotic, the right hand is not knowing what the left hand's doing and at the end of the day it's the child who suffers" (I12, mother of a child, SMA type 1)*
More integrative care and school places (e.g., in centres for chronically ill children, barrier-free schools)	• *"Grammar schools should please make sure that they are also disabled-accessible. (…) She's always been special anyway and now she's getting another spot because she can't go to the school where she'd like to, with her friends, just because she has a wheelchair (…)." (I25, mother of a child, SMA type 2-3)*
Close medical supervision and involvement in treatment planning	• *"Before this nusinersen administration, there was always a quarterly outpatient doctor's appointment, which no longer exists. With the medication, I have a gap in my knowledge, which should be clarified again in a doctor's consultation. (...) I'm always in email contact with them when something is wrong, but we've never had a direct doctor-patient conversation since then." (I9, mother of a child, SMA type 2)*

[a]original transcripts in German, quotes have been translated
